# A new methanogen “*Methanobrevibacter massiliense*” isolated in a case of severe periodontitis

**DOI:** 10.1186/s13104-017-2980-3

**Published:** 2017-12-01

**Authors:** Hong T. T. Huynh, Marion Pignoly, Michel Drancourt, Gérard Aboudharam

**Affiliations:** 1 UFR Odontologie, Aix-Marseille Université, 27, Boulevard Jean Moulin, Marseille Cedex 5, France; 2 URMITE, CNRS, UMR 7278, IRD 198, IHU Méditerranée-Infection, Aix-Marseille Université, Marseille, France

**Keywords:** “*Methanobrevibacter massiliense”*, *Pyramidobacter piscolens*, Periodontitis, Methanogen, Archaea

## Abstract

**Background:**

A few methanogens have been previously recovered from periodontitis lesions, yet their repertoire may not be completed. We recovered a previously unreported methanogen species in this situation.

**Case presentation:**

A 64-year-old Caucasian woman was diagnosed with chronic, severe generalized periodontitis. In the presence of negative controls, an 18-month culture of periodontal pockets in anaerobe Hungate tube yielded “*Methanobrevibacter massiliense*” and *Pyramidobacter piscolens*.

**Conclusions:**

This case report provides evidence of the symbiotic strategy deployed by the methanogens and the anaerobes, and reports the first culture of a new methanogen, “*M. massiliense*”.

**Electronic supplementary material:**

The online version of this article (10.1186/s13104-017-2980-3) contains supplementary material, which is available to authorized users.

## Background

Periodontitis is a multifactorial disease resulting in the progressive destruction of bone, formation of periodontal pockets and the progressive loss of function of teeth [1]. Complexes of various microorganisms have been implied in the genesis of periodontitis [2]. Archaea producing methane, i.e. methanogens recently emerged in these periodontitis microbial complexes [3]. More precisely, two methanogens *Methanobrevibacter oralis* and *Methanobrevibacter smithii* have been detected by culture [4] and culture-independent approaches [5], while a few other methanogens have been only detected by specific sequences [6]. “*Methanobrevibacter massiliense*” is one such yet uncultured methanogen that we consistently detected by investigating a large collection of 100 dental plaque specimens collected over five centuries in France (from 14th to 19th) (and previously named *Methanobrevibacter* sp. N13) [7]. We here report the first isolation of a new methanogen “*M. massiliense*” in mixed infection in a patient with severe periodontitis.

## Case presentation

**Fig. 1:**
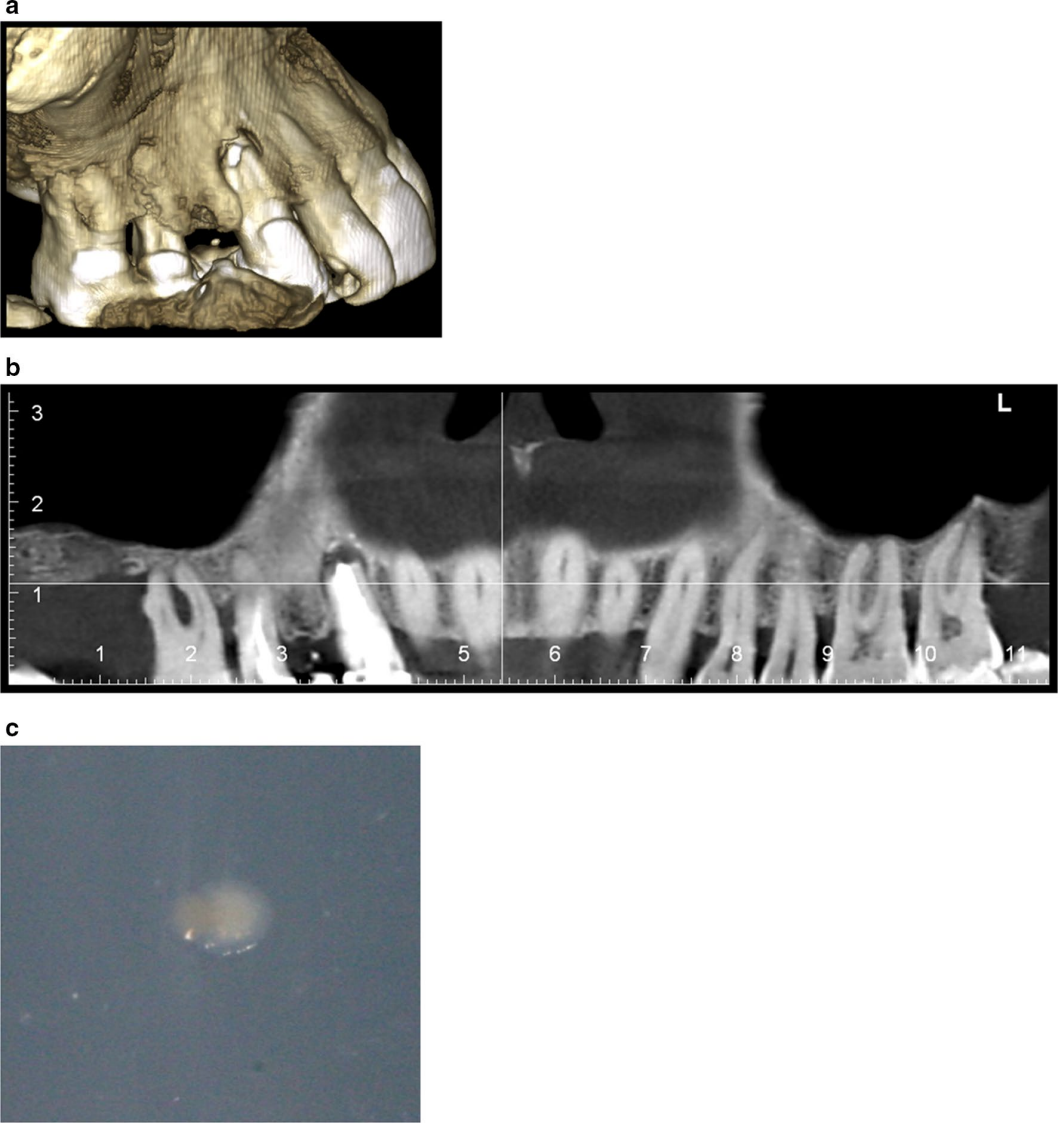
Severe periodontitis: Corn-beam aspect (**a**) and radiographic aspect (**b**) of a patient who yielded mixed anaerobe infection (**c** white colony, “*M. massiliense*”; pink colony, *P. piscolens*)

A 64-year-old Caucasian woman came to our Department for a dental consultation due to painful gums and mobile teeth. Her medical history was remarkable for asthma and tobacco smoking. Clinical examination showed generalized dental calculus, generalized bleeding on probing and pockets with a depth of 7 mm in tooth 38, 6 mm in teeth 16 and 27 and 5 mm in teeth 16, 15, 13, 12, 25, 26, 38, 37, 44 and 47. Radiography showed bone loss along the apex of 16 and up to the third center of 15 and 13–27. Teeth 13 and 15 showed apical infection and failed root canal treatment (Fig. 1). Chronic, severe generalized periodontitis was diagnosed and a dental plaque specimen was collected from teeth with deep pockets (teeth 16, 27 and 38) for microbial investigations after information of the patient and her consent. The sample was cultured under anaerobic conditions. Dental treatment consisted in scaling and root surface planning, restoration of teeth 11, 26, 34 and 35. After surfacing, pending temporary wound healing, mobile temporary prostheses were put in place. Maintenance and radiological monitoring were performed. Afterwards, the definitive mobile prostheses were realized. Follow-up in November 2015 found stable periodontal tissue, a second dental plaque specimen was collected from the remaining teeth with deep pockets and scaling-polishing was performed. In March 2016, tooth 16 was removed because of relapse, the movable prosthesis was modified accordingly and a third dental plaque specimen was collected from the remaining teeth with pockets.

## Materials and methods

Samples were collected from all periodontal pockets of the individual with sterile Gracey curettes 1/2 (Hu-Friedy, Rotterdam, Netherlands) and placed into Hungate tubes containing 5 mL of the SAB anoxic medium for methanogens composed of NiCl_2_·6H_2_O, 0.07 mg/L; FeSO_4_·7H_2_O, 0.2 mg/L; MgSO_4_·7H_2_O, 0.1 g/L; K_2_HPO_4_, 0.5 g/L; KH_2_PO_4_, 0.5 g/L; KCl 0.05 g/L; CaCl_2_, 0.05 g/L; NaCl, 1.5 g/L; NH_4_Cl, 1 g/L; NaAcetate, 1 g/L; yeast extract, 1 g/L; biotrypcase, 1 g/L; l-cysteine·HCl, 0.5 g/L; trace elements Widdel, 1 mL/L; resazurin, 1 mL/L; NaHCO_3_, 10%; Na_2_S, 2%; vancomycin, 100 mg/L, pH 7.5 with 10 M KOH (Signa-Aldrich, Lyon, France) [8]. The tubes inoculated with dental plaque and four negative control tubes containing non-inoculated medium were washed by a flux of nitrogen and were directly incubated at 37 °C with agitation under a mixture of 80% H_2_ + 20% CO_2_ at 2-bar pressure. Growth of methanogens was monitored by measuring methane production by using gas chromatography (Clarus 500, Perkin Elmer, Courtaboeuf, France). Tubes exhibiting methane production were then screened for *M. oralis* using a specific real-time PCR assay targeting the heat-shock protein *cnp*60 gene of *M. oralis* as previously described [9] (Additional file 1: Table S1). Distilled water was used as negative control. A Ct value of > 32 was considered as negative. Tubes negative for methane production were screened for the presence of methanogens using previously described partial PCR amplification and sequencing of the methyl-coenzyme M reductase (*mcr*A) gene [10] and the 16S rRNA gene [11]. The sequences were analyzed with the ChromasPro program, version 1.5, and similarity values were determined by BLAST program in the online analysis platform from NCBI (http://blast.ncbi.nlm.nih.gov). The *mcr*A and 16S rRNA gene sequence-based phylogenetic trees were analyzed with BLAST from NCBI. Further isolation of any methanogen from the Hungate broth tubes was performed according to the Hungate roll-tube method [12]. A 0.5-mL volume of broth collected from each Hungate tube in which methane had been detected, was transferred into a tube of 5 mL melted agar medium in the water bath of 50 °C and this tube was inverted to mix the inoculum. A serial dilution through eight tubes of agar medium was generated likewise. Roll tubes were obtained by rotating the agar medium under cold. These roll tubes were incubated using a gas mixture of H_2_/CO_2_ (80:20, v/v; at 2-bar pressure) at 37 °C in an upright position. Four non-inoculated, negative control tubes followed the same procedure.

In the presence of negative controls, an 18-month culture in a Hungate tube with methanogen medium and subculture on solid medium, the first dental plaque specimen collected from tooth no 16 yielded white colonies identified as “*M. massiliense*” by archaeal *mcr*A and 16S rRNA gene sequencing [13] and pink-orange colonies identified *Pyramidobacter piscolens* [14] by bacterial 16S rRNA gene sequencing (Fig. 1). After a 3-month incubation period, the second specimen yielded “*M. massiliense*” in liquid medium only, while the third specimen remained sterile after a 5-month incubation period. The presence of “*M. massiliense”* found from the first sample oriented the antibiotic therapy associated with the treatment of surfacing of the root surfaces. Treatment with metronidazole has been set up.

## Discussion

We here report on the first isolation of a new methanogen *“M. massiliense”* in one patient diagnosed with severe periodontitis. This case is further illustrating the symbiotic life of methanogen and an anaerobic bacterium here *P. piscolens* [15]. Periodontitis is characterized by the formation of tooth pockets leading to the loss of the tooth in the most severe cases [16, 17]. This disease is a prototype multifactorial disease implying anaerobe pathogens and host immune response [18]. Moreover, pathogens implied in periodontitis are forming bacterial complexes such as the red complex comprising *Porphyromonas gingivalis, Tannerella forsythensis* and *Treponema denticola* [2]. The inflamed tooth pockets become a chronic reservoir of bacteria, toxins and inflammatory mediators that can disseminate throughout the blood and lymph circulation and cause other infection in organism [19, 20]. Among anaerobe pathogens, the respective role of Bacteria and Archaea is not fully understood [21].

This long-term isolation of two very fastidious microorganisms cannot be trivial and this case report provides evidence of the satellitism strategy deployed by the methanogens (here, “*M. massiliense*”) and the anaerobes (here, *P. piscolens*) in periodontal pockets. Indeed, “*M. massiliense*” and *P. piscolens* were isolated together from the very same periodontal pocket; they were never isolated alone from any dental pocket; colonies were isolated in direct contacts; colonies from either organism were not isolated separately. We hypothesized that sulfate-reducing *P. piscolens* used CH_4_ released by “*M. massiliense”* to produce H_2_S; and that H_2_S could aggravate periodontitis lesions [17].

## Conclusions

Isolation of both “*M. massiliense”* and *P. piscolens* is illustrating the satellitism life of methanogens and an anaerobic bacterium [15].

## Additional file

**Additional file 1: Table S1.** List and characteristics of primers used in this work.
