# Supplementary material for: A new methanogen “Methanobrevibacter massiliense” isolated in a case of severe periodontitis
Source: BMC Res Notes. 2017 Dec 1;10:657. doi: 10.1186/s13104-017-2980-3 (PMC5710069; doi:10.1186/s13104-017-2980-3)
Supplement: Supplementary file 1 — Additional file 1: Table S1. List and characteristics of primers used in this work. [file 13104_2017_2980_MOESM1_ESM.docx]

**Table S1.**  List and characteristics of primers used in this work.

| Primer name | Sequence (5’-3’) | Length | Reference |
| --- | --- | --- | --- |
| mcrA LuF | GGTGGTGTMGGATTCACACARTAYGCWACAGC | 32 | (10) |
| mcrA LuR | TTCATTGCRTAGTTWGGRTAGTT | 23 | (10) |
| 16S 86F | GCTCAGTAACACGTGG | 16 | (11) |
| 16S 1340R | CGGTGTGTGCAAGGAG | 16 | (11) |
